# Supplementary material for: Time-series transcriptome analysis identified differentially expressed genes in broiler chicken infected with mixed Eimeria species
Source: Front Genet. 2022 Aug 8;13:886781. doi: 10.3389/fgene.2022.886781 (PMC9393255; doi:10.3389/fgene.2022.886781)
Supplement: Supplementary file 2 [file DataSheet1.ZIP › 4dpi_KEGG.Gsea.1625100298584/KEGG_METABOLISM_OF_XENOBIOTICS_BY_CYTOCHROME_P450.html]

Details for gene set KEGG\_METABOLISM\_OF\_XENOBIOTICS\_BY\_CYTOCHROME\_P450[GSEA]

|  || Dataset | TMM\_4dpi\_gct\_format\_4dpi\_gct\_format.Class\_4dpi.cls #PC\_versus\_NC.Class\_4dpi.cls #PC\_versus\_NC\_repos |
| Phenotype | Class\_4dpi.cls#PC\_versus\_NC\_repos |
| Upregulated in class | 0 |
| GeneSet | KEGG\_METABOLISM\_OF\_XENOBIOTICS\_BY\_CYTOCHROME\_P450 |
| Enrichment Score (ES) | -0.44591096 |
| Normalized Enrichment Score (NES) | -1.3932887 |
| Nominal p-value | 0.08238507 |
| FDR q-value | 0.32847372 |
| FWER p-Value | 0.996 |
Table: GSEA Results Summary

  

Fig 1: Enrichment plot: KEGG\_METABOLISM\_OF\_XENOBIOTICS\_BY\_CYTOCHROME\_P450      
 Profile of the Running ES Score & Positions of GeneSet Members on the Rank Ordered List

  

| SYMBOL | TITLE | RANK IN GENE LIST | RANK METRIC SCORE | RUNNING ES | CORE ENRICHMENT || 1 | DHDH | na | 106 | 1.605 | 0.0857 | No |
| 2 | CYP1B1 | na | 146 | 1.473 | 0.1692 | No |
| 3 | ALDH1A3 | na | 581 | 0.895 | 0.1857 | No |
| 4 | GSTT1 | na | 1493 | 0.521 | 0.1403 | No |
| 5 | GSTM2 | na | 2462 | 0.353 | 0.0804 | No |
| 6 | EPHX1 | na | 2980 | 0.285 | 0.0540 | No |
| 7 | MGST2 | na | 5918 | -0.001 | -0.1910 | No |
| 8 | GSTZ1 | na | 7777 | -0.159 | -0.3366 | No |
| 9 | CYP3A5 | na | 8491 | -0.228 | -0.3827 | No |
| 10 | GSTK1 | na | 9125 | -0.299 | -0.4179 | No |
| 11 | GSTO2 | na | 9462 | -0.343 | -0.4257 | Yes |
| 12 | CYP3A4 | na | 9602 | -0.362 | -0.4160 | Yes |
| 13 | ADH6 | na | 9642 | -0.367 | -0.3976 | Yes |
| 14 | UGT2A1 | na | 10045 | -0.421 | -0.4063 | Yes |
| 15 | MGST3 | na | 10072 | -0.424 | -0.3835 | Yes |
| 16 | ALDH3B2 | na | 10162 | -0.439 | -0.3651 | Yes |
| 17 | GSTA2 | na | 10745 | -0.548 | -0.3814 | Yes |
| 18 | MGST1 | na | 11272 | -0.692 | -0.3845 | Yes |
| 19 | GSTA3 | na | 11653 | -0.916 | -0.3623 | Yes |
| 20 | ADH4 | na | 11768 | -1.035 | -0.3109 | Yes |
| 21 | ADH1C | na | 11810 | -1.129 | -0.2478 | Yes |
| 22 | CYP2C18 | na | 11900 | -1.289 | -0.1793 | Yes |
| 23 | UGT1A1 | na | 11907 | -1.313 | -0.1025 | Yes |
| 24 | GSTA4 | na | 11979 | -1.885 | 0.0026 | Yes |
Table: GSEA details [plain text format]

  

Fig 2: KEGG\_METABOLISM\_OF\_XENOBIOTICS\_BY\_CYTOCHROME\_P450      
 Blue-Pink O' Gram in the Space of the Analyzed GeneSet

  

Fig 3: KEGG\_METABOLISM\_OF\_XENOBIOTICS\_BY\_CYTOCHROME\_P450: Random ES distribution      
 Gene set null distribution of ES for **KEGG\_METABOLISM\_OF\_XENOBIOTICS\_BY\_CYTOCHROME\_P450**
